# Academic and family disruptions during the COVID-19 pandemic: A reflexive from social work

**DOI:** 10.1177/1473325020973293

**Published:** 2021-03

**Authors:** Gabriela Rubilar Donoso, Caterine Galaz Valderrama, Catherine A LaBrenz

**Affiliations:** Departamento de Trabajo Social, Universidad de Chile, Santiago de Chile, Chile; School of Social Work, University of Texas at Arlington, Arlington, TX, USA

**Keywords:** Academia, transitions, narratives, self-interview, subjectivity

## Abstract

This article presents a reflection of how processes to reconcile work-life balance among academic mothers have changed during COVID-19. We present three autobiographical narratives that explore adapting and adjusting to research and teaching during remote work, confinement, and caring for one’s children. Intertwined in these narratives are themes of disruptions, responsibilities, and discoveries through these processes to adapt to COVID-19 and ongoing social and political crises.

## Conceptual framework

The transformation of formal social work over time has consisted of transition points that reflect evolving economic, social, and political contexts. These transition points have been researched and documented in countries with longstanding traditions of formal social work, such as the U.S. (Ruth and Marshall, 2017) and Chile (Saracostti et al., 2014). Furthermore, social work transformations have integrated interdisciplinary debates on issues such pedagogy and academia (Gray et al., 2015), gender equity and division of work (Domínguez et al., 2019), and processes of transformation and adaptation of work during times of crisis.

Traditionally, academic careers have followed a linear trajectory in which early career faculty progress to mid-career faculty and then to senior faculty, securing tenure along the way (McAlpine et al., 2014; Wolfinger et al., 2009). This linear trajectory is guided by evaluation criteria that universities apply, often related to research, teaching, and service expectations. Yet, these criteria often exacerbate disparities, such as gender disparities for academic who are also navigating motherhood (Wolfinger et al., 2009; Vásquez-Cupeiro and Elston, 2006).

This article explores the subjectivity of three academic-mothers’ experiences navigating work life, productivity, childcare, and uncertainty during the pandemic. We understand subjectivity within political, historical, and social contexts. In this paper, subjectivity is present in the significance that academic mothers place on their own experiences as they configure their identity through interaction with others and their surroundings. Through these processes, they are both a subject of and subjected to social structures and discourse. This approach is guided by the concept of social construction of subjectivity, through shared group experiences and understandings through which structural, intersubjective, and biographical dimensions intersect (Harding, 2006).

These narratives were written from a material-symbolic space: the “home,” within the context of confinement and merging work and family spaces. There are transformations in material and symbolic space when family space and work space are co-located (Bengtsson, 2006). Telework has an impact on gender disparities within traditional work settings, as it may provide more autonomy to balance other responsibilities (de Araújo et al., 2015). Although COVID-19 is still evolving, telework during the pandemic may exacerbate gender inequities instead of improving them. Indeed, gender inequities intersect with other differences such as child age and ability, individual processes of subjectivation, and lack of external social support among the three academic mothers whose narratives are presented in this article. All three of the academic mothers who present narratives have been directly impacted by the lack of support outside the home, be it extended family, schools, or other acquaintances who were more present before COVID-19. These narratives intertwine with inequalities to access services and supports; the two narratives from Chile reflect a country that has issued large-scale quarantine measures and prohibited contact between households. In contrast, the United States has left each state with autonomy to respond to the evolving pandemic. In Texas, some counties issued shelter-in-place orders and most public school districts closed, but many businesses, private schools, and childcare centers continue to function. Furthermore, there is no restriction on leaving one’s home and there is no restriction from socializing with others outside of one’s household.

We frame our narratives understanding of family and work experiences, in which these result from a triple construction based on overlapping individual, social, and institutional dimensions, such as relationships and meanings, and tensions that emerge in times of uncertainty.

## Context of each reflection

This paper is based on three narratives that were constructed by self-interviews (Boufoy-Bastick, 2004). The three participants were all tenure-track or tenured mothers in academia, each in a different phase of motherhood and academic trajectory. This is relevant as it reflects distinct moments in academic careers. They also had diverse types of family composition, with children in different stages of development, and differing types of external support. Geographically, two of the narratives were constructed in Chile and one in the United States, but the experiences of the three overlapped and had some shared circumstances due to remote work, confinement, and dual identity of academic and mother.

The first narrative presents a reflection from a junior tenure track academic mother who works at a large, public research-intensive university in the Southern United States. Research-intensive universities in the U.S. are guided by principles of productivity, often measured by scholarship and funding. There have been few state- or nation-wide initiatives or policies to support women in academia who are also mothers, although some individual universities or departments (such as the one from the first narrative) have offered flexibility in teaching modality and tenure clock extension to accommodate academic mothers. Notably, in Texas childcare centers have mostly remained open, placing the responsibility for decision-making regarding risk and exposure on each family. In contrast, the Chilean government has forced all public and private childcare centers to close temporarily due to COVID-19.

The second and third narratives come from academic mothers in Chile, where higher education is heavily guided by neoliberalism and principles of excellence and productivity. In Chile, the government has little control over family dynamics as most family policy has focused on economic production. This has been particularly relevant during the current pandemic, as policies to support families have focused on economic assistance, without recognizing gender roles and responsibilities. As such, the triple shift that many academic mothers were experiencing before the pandemic has intensified and become more evident, resulting in individual and social consequences.

Three academic mothers engaged in processes of reflexivity to deconstruct the disruption of COVID-19 on their domestic and academic work and family time (Cornejo et al., 2019; Rubilar, 2013, 2015). To construct narratives from the reflexive processes, the authors followed the directives of the narrative-biographical approach that focus on trajectories (Rubilar, 2017).

## Three short reflections

### COVID-19 as a fisruption to M.’s daily routine

It’s 9:30 pm on day 74 of quarantine. I take a deep breath as I decide how to spend the last few hours of my day before I sleep. Tomorrow M turns three. There are balloons to inflate, streamers to hang, presents to wrap. But there are also emails to answer, papers to revise, and proposals to finish. Since early March, my partner and I have done the same dance—wake up, work, watch M., trade, repeat. This is officially the longest we have spent together—even longer than when M was born, as she started daycare at 11 weeks.

As a researcher of family resilience and maltreatment, every step on my journey to parenthood was carefully planned, constructed to allow for maximum flexibility and balance. But, as with motherhood in general, COVID has shown us that as well laid-out as plans can be, life happens. We must be flexible, adapt, and learn that it is okay to take a break.

Yet, there is concern that mother academics are decreasing productivity during these times, especially when compared to male colleagues. I’ve long given up trying to have meetings, write, or complete any other task beyond emails while it is my shift with M. Her preschool is still open, but with thousands of new cases per day in our part of Texas, we must consider the risk of sending her back to school. So for now, we continue with our bandaid approach. Even if she were able to entertain herself (which she is not), I think back to my work as a social work practitioner with young children who had experienced maltreatment. After working with extreme cases of neglect, how could I in good conscience conduct research and write articles on parent-child attachment and parenting, all the while neglecting the development and stimulation of my own child?

I remind myself that emails can be answered tomorrow. Revisions can be sent a day late. The proposal can be submitted later in the evening, after M. has gone to sleep. What can’t be postponed? M’s laughter as we chase one another. Her excitement as she learns to draw another letter. And, my own amazement at just how quickly she grows. For now, this is exactly where I am meant to be. As a mother, a social worker, and a tenure-track academic, this is what I do. I juggle. I multi-task. I focus my research parent-child interaction and resilience. But for now, I will prepare for M.’s birthday. The rest can wait.

### Distractions from the weight of the triple shift without support

I am quarantining in a small apartment with my 6-year-old son. I am separated, so that means that the majority of child care and domestic responsibilities fall on me. The most difficult thing has been to fit in remote work, school work, therapies that he needs to have four times per week, since my child has a disability, as well as cleaning, making food, and other household chores. There are days I am on the brink of exhaustion. I see colleagues writing articles and catching up on reading, while I can barely read two pages in a row.

The first few weeks of quarantine were particularly hard, because the government did not give parents permission to go out with their children. I made it two weeks and finally we were able to get permission to go out with B., and that way I had at least two or three days a week without having to watch him constantly. Our society implicitly assumes that mothers should be responsible for their children. I feel completely alone. I have asked for help from my family and some external paid support, but everyone is scared and no one wants to leave their house.

Each day is the same routine: answering emails, making food, teaching classes, and setting some hours aside for B.’s homework, then cooking again, cleaning up, B.’s physical therapy, meetings … .I feel depressed, tired, and stressed out.

Perhaps at another point in time when families were not so nuclear, rather extensive, childcare would have been different. But, we are living in the present and I think that individualism has been much more prevalent in this pandemic than solidarity. I try to stay positive: I do exercise with my son, every now and then I get together with Friends online. From time to time I see the positive side of quarantine: more hugs from my son, listening to how he creates stories with his toys. These are the small parentheses among our confinement.

### Discovering a new way of inhabiting domestic space

The current confinement has me disconcerted as I found myself without repertoire in an apartment that I still have not finished furnishing. As a result, it felt distant and unfamiliar at the beginning. Today, over the course of 120 days of confinement, it has become my own, with its own sounds, dynamics, and neighbors. What has been the hardest for me is to reorganize my work. Initially, I would spend entire days having virtual meetings with university leadership and faculty, all the while having less time for my research projects.

Staying at home, for someone who tended to never be at home, has not been easy. I have had to deal with issues of separation and caring for my teenage children, cooking for them when they visit, and cleaning without obsessing over it. Since the first week I have had regular contact with students through virtual meetings and with them we have learned to use the platforms, to adjust the rhythm, and to optimize the use of the network. I did not have internet at home, so I had to contract internet for my apartment and I bought wireless headphones to be able to move around more, since the hours upon hours of sitting were doing a number on my knees.

The time in quarantine has created a loop that takes me to the past. I have thought a lot about my undergraduate education and the experience that my students are having now, and how this situation will stay with them for the rest of their lives. Living alone and being alone for several days each week has been an unprecedented experience for me at my 47 years of life. It is the first time that I spend so much time without seeing or talking to anyone. Most of the time I enjoy it, although there are days that seem eternal.

On one of his visits, my teenage son brings me a radio, which I appreciate because listening to it allows me to feel accompanied. I often fall asleep while listening to talk shows or music. Radio was very present throughout my own childhood and I spent my entire adolescence with the dial tuned. From that time, I remember the dictatorship and our hope placed on a political change radically different from the situation that was going on around us … Today the radio broadcasts news of the curfew, the military and police controlling the quarantined areas, all constantly remind me of the fragility of our lives, of our democracy, and the need to have a more profound change in our institutions. Changing, transforming, and adapting has been a constant during this period of enclosure.

## Discussion

The experiences of confinement and telework during COVID-19 have been particularly detrimental for academic mothers and their trajectories. They are not just witnessing their careers and metrics lag behind those of male colleagues or other females without children; they are also compromising their mental and physical health by trying to “do-it-all” through a triple shift of productivity, housework, and childcare

The shift to online learning has also resulted in technological and generational adjustments, especially evident among older generations. The use of digital platforms, video recording and editing, and the transition to telework have also highlighted generational inequities.

In parallel, colocation of work and family spaces during COVID-19 has erased limits and boundaries that many constructed to separate their public and private worlds. The private lives of academics are made public by the use of online platforms; opinions they may share on social networks and communication within their own homes may inadvertently be shared among students and colleagues.

While we have observed gender differences and inequities in the triple shift of academic mothers, we have also witnessed opportunities for collaborative networks and mutual help. Together, these three narratives reflect diverse situations yet shared experiences of motherhood and academia in times of confinement, online teaching, reflexivity and resistance to notions of individualism that offer opportunities for discoveries of camaraderie and support.

## Concluding remarks

Over the course of several months of the COVID-19 pandemic, this paper reflects how we have attempted to find balance between our work and family responsibilities during quarantine, while confronting interruptions and disruptions. We have had to and continue to adapt our ways of conducting research and teaching, to integrate the changing contexts in which we immerse our work. As we transition to a new school year and semester, we must continue to examine how our context and surroundings not only impact our own work, but also the subjects we work with, our students, and our children, all of whom are experiencing their own similar, yet unique, transformations.
